# Complication of colo-colic intussusception following colonoscopy: A case report and review of literature

**DOI:** 10.1016/j.ijscr.2025.111755

**Published:** 2025-07-29

**Authors:** Sophie Rienecker, Thampi Rawther

**Affiliations:** Wagga Wagga Base Hospital

**Keywords:** Intussusception, Colonoscopy, Complication, Case report

## Abstract

**Introduction and importance:**

Colonic intussusception is a rare complication following colonoscopy. This case report describes an unusual instance of colo-colic intussusception after endoscopic removal of polyps, highlighting the importance of post-procedural monitoring.

**Case presentation:**

A 58-year-old female presented with cramping abdominal pain, rectal bleeding, and fever 15 hours after a routine colonoscopy. CT imaging revealed a ‘target sign’ consistent with colo-colic intussusception.

**Clinical discussion:**

Management of post-colonoscopy intussusception varies from conservative to surgical approaches. In this case, repeat colonoscopy confirmed spontaneous resolution, thus avoiding surgery.

**Conclusion:**

This case highlights the need for clinicians to maintain a high index of suspicion for rare but significant complications such as intussusception following colonoscopy. Prompt recognition and appropriate follow-up are essential to guide management and improve patient outcomes. It is important to consider risk factors that may increase a patient's risk of developing intussusception.

## Highlights

• **Rare complication**: Colonic intussusception following a routine colonoscopy for surveillance.
• **Post-procedural symptoms**: Abdominal cramping, rectal bleeding, and fever.
• **Timely diagnosis**: Repeat colonoscopy revealed a resolved colo-colic intussusception.
• **Management approach**: Conservative management with repeat colonoscopy and IV antibiotics led to symptom resolution
• **Educational value**: Highlights the importance of considering rare complications like intussusception in patients presenting with post-colonoscopy symptoms

## 1 Introduction

Intussusception is a rare but documented complication following colonoscopy, typically resulting from polypectomy or manipulation during the procedure [1]. This report presents a case of colonic intussusception in a patient undergoing routine surveillance colonoscopy for previous polyps. Although colonic intussusception is unusual post-colonoscopy, early recognition and appropriate management are crucial to avoid severe complications. This case report has been reported in line with the SCARE criteria [2].

## 2 Case presentation

A 58-year-old female with a medical history of rheumatoid arthritis, Sjögren's syndrome, Felty's syndrome, bronchiectasis, and serrated colonic polyps underwent a routine surveillance colonoscopy on 17/7/24 due to previous sessile polyps. The procedure was uncomplicated, with a single polyp removed from the transverse colon using a cold snare after lifting. Immediately post-procedure, the patient was pain-free and discharged without any issues. Despite the co-morbidities of this patient, she was generally well; her auto-immune conditions were well controlled.

After returning home, the patient began experiencing cramping, intermittent abdominal pain, initially severe but gradually improving. She also noted a single episode of bright red rectal bleeding. She presented to the emergency department 15 hours after the colonoscopy was completed.

Upon presentation to the hospital, the patient was febrile at 38.5 °C, her heart rate and blood pressure were within normal limits. Physical examination revealed tenderness in the epigastric region, but the abdomen remained soft. Assessment for differential causes of her fevers revealed a clear chest, no urinary symptoms, and no other overt signs of infection elsewhere. There were no signs of a flare of her rheumatoid arthritis or exacerbation of her bronchiectasis. Laboratory investigations revealed:

• White cell count (WCC): 8.1 × 10^9/L
• C-reactive protein (CRP): 1.0 mg/L
• Lactate: 2.1 mmol/L.

A contrast-enhanced CT of the abdomen and pelvis (CTAP) demonstrated a classic ‘target sign’ supporting the diagnosis of large bowel intussusception involving the transverse colon. A differential diagnosis included post-polypectomy haematoma. She was admitted under surgical care for further management.

## 3 Clinical course

**Fig. 1:**
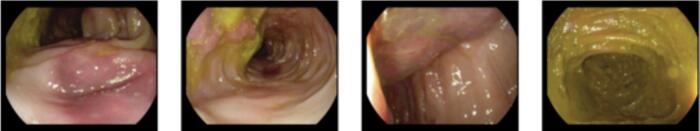
Images taken during repeat colonoscopy.

The following day, the patient underwent a repeat colonoscopy performed by the general surgical consultant. At 60 cm from the anal verge, a small area of bleeding was observed at the proximal polypectomy site. 20 cm beyond this, an area likely indicative of a resolved colo-colic intussusception was found to be erythematous and oedematous, refer to Fig. 1. This confirmed the provisional diagnosis of intussusception and supported that the CT findings were not due to a haematoma.

The patient remained admitted for 3 days, during which her pain gradually improved, and she was able to tolerate oral intake. Despite persistent fever, she showed no further signs of infection. The patient received 3 days of intravenous antibiotics, which were discontinued once the fever subsided and no further complications were noted. Her complex medical background of autoimmune conditions such as rheumatoid arthritis and Sjögren's Syndrome were considered, it was queried whether the this autoimmune panel impacted the connective tissue of her GIT and increased her risk of this complication.

## 4 Discussion

Intussusception in adults is rare and is typically associated with pathological aetiology. Symptoms such as abdominal pain, cramping, rectal bleeding, and fever, all of which were observed in this case, may present in intussusception [1,5].

Intussusception after colonoscopy is uncommon, with only 13 documented cases. The most common symptom observed in other cases was abdominal pain post-colonoscopy. Diagnosis can be aided by CT imaging, which may reveal the ‘target sign’—the centre of the intussusceptum and the external ring formed by the intussuscipiens [1].

Previous cases of post-colonoscopy intussusception have been potentially linked to colonoscopic manipulation or complications post-polypectomy. Polypectomy and scope withdrawal may contribute to intussusception via transient changes in intraluminal pressure or mucosal traction; one theory suggests that a vacuum is created as the scope is withdrawn, causing collapse and invagination of the bowel [3,11]. In this case, cold-snare polypectomy may have created localized mucosal edema, facilitating invagination.

This case also raises discussion around risk factors for developing intussusception post colonoscopy. This patient had a complex medical background with autoimmune conditions of rheumatoid arthritis, Felty's Syndrome and Sjögren's syndrome. The connective tissue of the GIT could be impaired when these conditions are present and could increase a patient's risk of intussusception. GIT involvement of rheumatoid arthritis involves diminished peristalsis and thick collagen in the colonic wall [4].

Management of previous cases of intussusception post-colonoscopy has varied, given the low number of cases reported there is not a clear treatment strategy and hence increasing the literature can aid to guide appropriate management. Majority of cases the patients underwent surgery (63 %) [3]. The surgeries included: laparoscopic reduction, with one requiring intra-operative colonoscopy to assess colon mucosa viability; one patient required laparoscopic ileocolic resection; four underwent laparotomy, of which three requiring right hemicolectomy and one ileocolic resection. One patient underwent a repeat colonoscopy that was reassuring for no intussusception [1,[6], [7], [8], [9]]. The management in this case involved a repeat colonoscopy and confirmation of resolved intussusception.

Management of adult intussusception in general also varies, with surgical management typically preferred due to the high likelihood of malignancy being the causal factor. Often, management involves an exploratory laparotomy or laparoscopic procedure with potential resection of lead point masses or areas of ischaemia [3,10].

Early recognition of this complication is crucial to avoid more severe outcomes. Though the symptoms can be vague, tools such as CT imaging are helpful in diagnosis. Given the rarity, increasing documentation of such cases is essential to help guide future management protocols.

## 5 Conclusion

This case illustrates the importance of close monitoring after a colonoscopy. While post-procedural complications like intussusception are rare, they should be considered in the differential diagnosis for patients presenting with abdominal symptoms following colonoscopy. Timely diagnosis through imaging and consideration of interventions such as surgery or repeat colonoscopy, when necessary, is essential for managing such complications.

## CRediT authorship contribution statement

Sophie Rienecker and Thampi Rawther – case report idea
Sophie Rienecker – writing of report
Thampi Rawther – final edit and overview.

## Ethical approval

Ethics approval was not required as this is a case report.

Written informed consent was obtained from the patient for publication of this case report and accompanying images.

## Guarantor

Sophie Rienecker
Thampi Rawther.

## Funding

No funding was required for this case report. The authors have no relevant financial or non-financial interests to disclose.

## Declaration of competing interest

The authors have no conflicts of interest to declare that are relevant to the content of this article.

## References

[1] Hassan W.A.W., Teoh W. (2018). Intussusception after colonoscopy: a case report and review of literature. Clinical Endoscopy.

[2] Kerwan A., Al-Jabir A., Mathew G., Sohrabi C., Rashid R., Franchi T., Nicola M., Agha M., Agha R.A. (2025). Revised Surgical CAse REport (SCARE) guideline: an update for the age of artificial intelligence. Premier Journal of Science.

[3] Ligato I., Ruffa A., Sbarigia C., Petrucciani N., Esposito G. (2024). Surprising complication of intussusception after colonoscopy. A case report and a review of the literature. Journal of Surgery..

[4] Cojocaru M., Cojocaru I.M., Silosi I., Vrabie C.D. (Jan 2011). Gastrointestinal manifestations in systemic autoimmune diseases. Maedica (Bucur)..

[5] Brill A., Lopez R.A. (2023). StatPearls.

[6] Ho M.M., Park J.J., Prasad L.M. (2010). Post colonoscopy colonic intussusception reduced via a laparoscopic approach. JSLS: Journal of the Society of Laparoendoscopic Surgeons.

[7] Luciano E., Marar O., Cocco M. (2022). Colocolonic intussusception of a colostomy after colonoscopy. Journal of Surgical Case Reports.

[8] Lasithiotakis K., Grisbolaki E., Filis D., Athanasakis I., Zoras O., Chalkiadakis G. (2012). Ileocolic intussusception precipitated by diagnostic colonoscopy: a case report. Surg. Laparosc. Endosc. Percutan. Tech..

[9] Min M.X., Sklow B., Vaughn B.P. (2017). Intussusception after routine colonoscopy: a rare complication. ACG Case Reports Journal.

[10] Nachnani J., Burns E., Margolin D., Clarkston W.K. (2012). Colocolonic intussusception after colonoscopy. Gastrointest. Endosc..

[11] Xiang S.H., Xu G.Q. (2024). Colo-colonic intussusception as a rare complication of colonoscopy with polypectomy: two case reports. World Journal of Gastrointestinal Surgery.

